# Comprehensive assessment to reveal the salt tolerance potential of cultivated eggplants and their wild relatives

**DOI:** 10.3389/fpls.2025.1483409

**Published:** 2025-01-30

**Authors:** Esra Cebeci, Hatice Filiz Boyaci, Sevinc Kiran, Sekure Sebnem Ellialtioglu

**Affiliations:** ^1^ Department of Vegetable Breeding and Ornamentals, Bati Akdeniz Agricultural Research Institute, Antalya, Türkiye; ^2^ Department of Horticulture, Faculty of Agriculture, Recep Tayyip Erdogan University, Rize, Türkiye; ^3^ Department of Horticulture, Faculty of Agriculture, Ankara University, Ankara, Türkiye; ^4^ Ankara University Technopolis, Doqutech Academy LLC. Co., Golbasi, Ankara, Türkiye

**Keywords:** eggplant, correlation, crop wild relatives, malondialdehyde, PCA, proline, salt stress

## Abstract

This study aimed to uncover salt-tolerant eggplant (*Solanum melongena* L.) genotypes and accessions. Crop wild relatives (*S. macrocarpon* L., *S. linnaeanum, S. incanum* L., *S. insanum* L., *S. sisymbriifolium* Lam.), commercial varieties (Topan374, Kemer, Amadeo, Faselis, Bildircin), and local genotypes (TB, BB, MK, AH) were investigated under 150 mM NaCl stress. The experiment was set in a completely randomized block design with three replications. Morphological and biochemical parameters were studied to distinguish salt-tolerant genotypes. Wild relatives have species-specific growth features; thus, the salt tolerance levels of morphologic features such as plant height and leaf area were found inappropriate to be compared. In eggplant, Na^+^ is a majorly harmful ion and there is a negative correlation between leaf Na^+^ content and plant tolerance index. The low Na^+^ concentration in roots of BB and S. linnaeanum caused high K^+^ and Ca^2+^ concentrations in their leaves. A plant with high proline accumulation displays greater tolerance under stress conditions. The proline content of *S. linnaeanum, S. incanum* L., and MK was analyzed to be higher than the others. Additionally, the lowest malondialdehyde (MDA) increases were observed in *S. linnaeanum*, TB, and *S. incanum* L. Moreover, positive correlations were spotted between 0-5 scale values and MDA and Na^+^ level in shoots by correlation analysis. Strong correlations between proline accumulation–S. linnaeanum and MDA accumulation–AH were revealed by principal component analysis (PCA). In terms of results, the most salt-tolerant, *S. linnaeanum, S. incanum* L., BB, and MK, will be employed in future breeding studies to improve salt-tolerant inbred lines and varieties through interspecific hybridization.

## 1 Introduction

Sustainable agriculture is under threat of biotic and abiotic stresses. One of them is salt stress, which is an urgent threat to vegetable production in arid regions, semiarid regions (Hannachi and Van Labeke, 2018), and greenhouses around the Mediterranean climate zone. Salinization mostly occurs due to the high evaporation and lack of rain; salts which were added by fertilization could not wash away and accumulate in soil. Minhas et al. (2020) emphasized that high salt concentration around the root zone prevents the water and mineral uptake and decreases the plant growth. Another salinity effect on plant metabolism has been associated with the accumulation of toxic ions (such as, Na^+^, Cl^-^, SO_4_), which causes alterations of the enzyme activities, photosynthesis, and protein synthesis (Zheng et al., 2008). When exposed to salt stress, plants respond through a number of ways, which could be used to determine tolerance or sensitivity. One of them is level of MDA, which is the end product of lipid peroxidation and a potential indicator of oxidative stress. The other is proline, as an osmolyte, which plays a major role to protect plants from negative effects of salt stress (Sairam and Tyagi, 2004). Although there have been many studies dealing with techniques to reduce detrimental effects of salt stress, there are few publications evaluating different local cultivars and wild relatives of eggplant for their salt tolerance.

Salt stress reduces the yield and restricts the agricultural production. The use of resistant cultivars on impacted lands is an environmentally friendly solution. An eggplant cultivar, its wild relative *Solanum insanum*, and their interspecific hybrid were grown under 200-mM and 400-mM NaCl doses. The hybrid exhibited more tolerance than either of two parents in terms of growth parameters, such as plant height and fresh weight. These results suggested the possibility of developing new salt-tolerant inbred lines or varieties via introducing genes from *S. insanum* (Ortega-Albero et al., 2023). Eggplant varieties under salt stress did not exhibit the same reactions (Yasar et al., 2013; Hannachi and Van Labeke, 2018; Hannachi et al., 2022). Finding variations among the genotypes in advance could support breeding efforts to improve salinity tolerance of eggplant varieties (Knapp and Vorontsova, 2016; Brenes et al., 2020a). Maas (1990) described the eggplant as moderately sensitive to salinity stress. The threshold values for salt tolerance were reported to be 1.5 dS m^−1^ for fruit yield (Unlukara et al., 2010) and 6.7 dS m^−1^ for shoot biomass (Heuer et al., 1986). Thus, it is urgent to improve resilient cultivars to sustain production under stressed lands. Previously, new varieties have been improved considering high yield and major pests and diseases; however, resilience to major abiotic stresses such as drought and salinity was mostly ignored. These breeding studies also caused the reductions in genetic diversity. Local genotypes, as a crucial gene source to enhance tolerance to abiotic stresses, may play an important role on reducing the effects of salt stress (Suarez et al., 2021). Eggplant has significant wild relatives which are known to have more tolerance to abiotic stresses, and it can be crossed with many of them (Plazas et al., 2019). After interspecific hybridization, introgression breeding through backcrossing with *S. melongena* helps in insertion of some tolerance features of wild relatives into the eggplant gene pool (Kouassi et al., 2016; García-Fortea et al., 2019).

Literature generally focuses on finding the effects of different salt stress degrees on commercial varieties and alleviating the salt effects on plants. However, this study was conducted to reveal salt-tolerant eggplant genotypes and accessions to employ in future breeding programs. A dose of 150-mM NaCl application was determined as an effective dose to select salt-tolerant individuals from previous studies of the researchers preferred.

## 2 Material and methods

### 2.1 Plant material and experiment area

A total of 14 eggplant genotypes and accessions from different geographic regions consisted of crop wild relatives (CWRs), which originated from continental Africa, local genotypes and commercial varieties were comparatively investigated under 0-mM and 150-mM NaCl doses to reveal their salt tolerance levels. Information regarding the plant materials is presented in **Table 1**.

**Table 1:** Codes, species names, and providing sources of the eggplant accessions.

Code/name	Species name	Type	Providing source
MM132	*S. macrocarpon* L.	Wild	INRAE, France
MM195	*S. linnaeanum* Hepper & Jaeger	Wild	INRAE, France
MM684	*S. incanum* L. group C	Wild	INRAE, France
MM510	*S. insanum* L. group E	Wild	INRAE, France
*S. siymbriifolium*	*S. sisymbriifolium* Lam.	Wild	INRAE, France
TB*	*S. melongena* L.	Local genotype	BATEM, Türkiye
BB *	*S. melongena* L.	Local genotype	BATEM, Türkiye
MK*	*S. melongena* L.	Local genotype	BATEM, Türkiye
AH*	*S. melongena* L.	Local genotype	BATEM, Türkiye
Topan374	*S. melongena* L.	Commercial variety (OP)	Asgen Seed Co.
Kemer	*S. melongena* L.	Commercial variety (OP)	Asgen Seed Co.
Amadeo	*S. melongena* L.	Commercial variety (F_1_)	Enza Zaden Seed Co.
Faselis	*S. melongena* L.	Commercial variety (F_1_)	Bayer group Co.
Bildircin	*S. melongena* L.	Commercial variety (F_1_)	BT Seed Co.

*These local genotypes belong to the BATEM eggplant gene pool, collected, and inbred to the F_6_ level.

The seeds were sown in viols (15 × 10) containing peat moss and perlite (1:1/v:v). Thirty days after sowing, healthy seedlings with two to three true leaves were transplanted individually to 1-L pots filled with a mixture of peat moss and perlite (1:1/v:v). All the pots were irrigated equally with Hoagland nutrient solution (Hoagland and Arnon, 1950) during 2 weeks until achieving sufficient root development. The study was conducted in semi-controlled greenhouse conditions. During the experiment, the temperature (°C) and relative humidity (%) of the greenhouse were recorded by the Hobo data logger (Apogee instruments, US).

### 2.2 Experimental design, stress treatment, and symptom observation

The experiment was set in a completely randomized block design with three replications, and each replication had six pots with one plant per pot. Additionally, a set of the same quantity plants for each genotype was used as control. All plants were used in phenotypic measurements. Because it is able to determine the salt tolerance of eggplants in a short time, the dose of 150 mM NaCl was chosen considering previous studies (Akinci et al., 2004; Assaha et al., 2013; Hannachi and Van Labeke, 2018; Brenes et al., 2020a; Brenes et al., 2020b; Alkhatib et al., 2021; Cebeci et al., 2024). When the seedlings in pots reached to the four–five true leaf stage, salt stress was applied until the final concentration of 150 mM NaCl was completed. At the same time, control plants were irrigated with an equal amount of non-saline water. After the final salt treatment, we waited for 12 days which is also the end of the experiment time for symptoms to emerge (Assaha et al., 2015; Alkhatib et al., 2021; Habila et al., 2021; Cebeci et al., 2024); symptom severity was compared with the control group using the 0–5 visual damage scale suggested by Kiran et al. (2015), where 0 denotes no effect, 1 denotes local yellowing and curling of leaves and slow growth, 2 denotes necrosis and chlorosis on 25% of the leaf, 3 denotes necrotic spots on the leaves and defoliation by 25%–50%; 4 denotes necrosis by 50%–75% and death of several plants, and 5 denotes formation of severe necrosis on leaves by 75%–100% and/or predominant deaths in plants.

### 2.3 Plant growth and water content

Plant height and leaf area were measured when the seedlings were alive. For the determination of plant height, all plants were measured from the soil ground to the shoot tip using a ruler, and the area of the top third leaf of each plant was determined using a portable leaf area meter (LI-3100, LI-COR, Lincoln, NE) on the 12th day of salt treatment. Later, shoots and roots were separated using scissors and weighed with a scale (0.01 g accuracy) to obtain the root and shoot (leaves + stem) fresh weight of each replication of every treatment (Kirnak et al., 2002; Özkay et al., 2014; Cebeci, 2024). Root and shoot (leaves + stem) samples were dried at 65°C for 48 h to constant weight and weighed again for defining dry weights (Kirnak et al., 2002; Cebeci, 2024). Dried root and shoot samples were also used for ion analysis. Lastly, the top third vigorous leaf of each plant was collected and stored in −20°C to MDA and proline analysis. Water content (WC) of shoot and root was calculated using fresh weight (FW) and dry weight (DW) values by Equation 1 (Cheng et al., 2018; Plazas et al., 2019; Cebeci, 2024):


(1) WC (%)=((FW−DW)/FW)×100

### 2.4 Na^+^, K^+^, and Ca^2+^ ions, MDA, and proline quantification

Dried shoot and root samples powdered before wet ashing in HC1O_4_:HNO_3_ (4:1 v:v) solution and ion accumulation were determined by an atomic absorption spectrophotometer (PerkinElmer 3100, United States) (Jones and Case, 1990). MDA and proline accumulation was analyzed from fresh leaf tissues by the trichloroacetic/thiobarbituric acid method (Hodges et al., 1999) and the ninhydrin-acetic acid method (Bates et al., 1973), respectively, using the spectrophotometer and expressed as μ mol g^−1^ FW.

### 2.5 Electrical conductivity of substrate

When the experiment was finished, all plants were removed from the pots for analysis, and the remaining substrate was dried. Later, a soil/water (1:5) suspension was prepared in deionized water and mixed for an hour with a shaker. Electrical conductivity (EC) was measured using a portable conductivity meter MW302 (Milwaukee Instruments, USA) and expressed in dS m^−1^.

### 2.6 Data analysis

Statistical evaluation of the data for drought stress tolerance was performed by analysis of variance test utilizing the JMP 7.0 software package (SAS Institute, Cary, NC, United States of America). Differences among the mean values were calculated with the LSD test at *p* < 0.001, *p* < 0.01, and *p* < 0.05. Besides the statistical evaluation, the percent rate of change (%) values of all measured traits under salt stress effect was calculated via Equation 2 (where ST: salt treated; C: control) and presented as Table (**Supplementary Table S1**) for the better understanding of the genotype’s tolerance level.


(2) Percent change (%)=[(ST−C)/C]×100

Relations between parameters were revealed using principal component analysis (PCA) by the MS Excel XLSTAT program. The correlation between the investigated features is presented with the correlation diagram, and a polar heat map was created to demonstrate hierarchical clustering between all morphological and physiological datasets by using OriginPro 2024 (OriginLab 264 Corporation, USA) software.

## 3 Results

Screening of existing germplasm and breeding of tolerant pure lines and varieties are among the major strategies to combat the salt stress effects. In this study, some plant growth features and biochemical parameters were assessed to determine the tolerance degrees of different eggplant genotypes and accessions to applied salt stress. During the experiment in a greenhouse, temperature and relative humidity ranged between 15°C and 42°C and between 23% and 72%, respectively, and the mean values recorded were 25.1°C and 39.2%, respectively (**Supplementary Figure S1**). Ors and Suarez (2016) announced that salt-stressed plants exhibit more damages under higher temperatures. During the experiment, temperatures which were above for eggplant’s optimum growth (22°C–30°C) were recorded. When the experiment was finished, the mean electrical conductivity (EC) of the used substrate was determined as 0.75 dS m^−1^ for control and 7.85 dS m^−1^ for salt treated group, respectively. Eggplant is known to be moderately sensitive to salt stress (Maas, 1990; Unlukara et al., 2010); thus, created stress conditions for the experiment were suitable to reveal the tolerance capacity of the employed genotypes and accessions.

**Figure 1:**
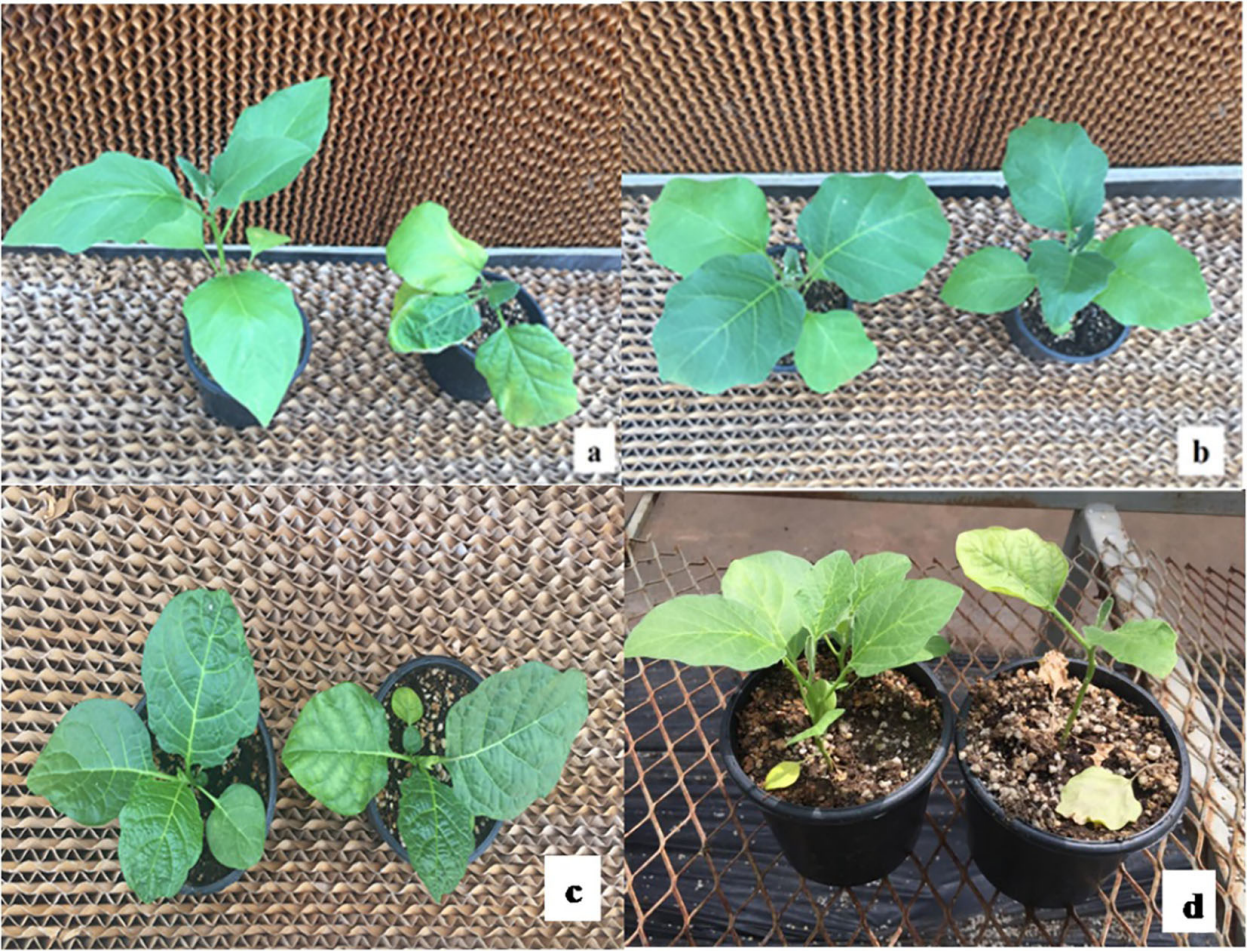
The phenotypic appearance of some genotypes (**A:** AH, **B:** MK, **C:** MM132, **D:** MM510, control plants on the left, salt-stressed plants on the right).

On the 12th day, after the final salt treatment, responses of the genotypes were examined phenotypically using a 0–5 visual scale (Kiran et al., 2015). While the seedlings of the control group were accepted to have no symptom and got “0,” the levels of symptoms induced by salt stress on the genotypes were found different from each other and the mean scale values ranked between 0.27 and 3.20 (**Table 2**). The phenotypic variations of some genotypes in response to salt stress are presented in **Figure 1** (Detailed pictures presented in **Supplementary Figure S2**).

**Table 2:** The plant height, leaf area, shoot fresh weight, shoot dry weight, root fresh weight, root dry weight and scale evaluation of eggplant genotypes at 12^th^ day of the 150 mM NaCl treatment*.

Genotype	Plant Height (cm)		Leaf Area cm^2^ plant^-1^		Shoot Fresh Weightg plant^-1^		Shoot Dry Weightg plant^-1^		Root Fresh Weightg plant^-1^		Root Dry Weightg plant^-1^		Scale-evaluation	
	Control	150 mM NaCl	Control	150 mM NaCl	Control	150 mM NaCl	Control	150 mM NaCl	Control	150 mM NaCl	Control	150 mM NaCl	Control	150 mM NaCl
**MM132**	10.50±1.87^k-m^	7.40±1.06^no^	71.86±11.32^a^	49.33±14.76^fg^	8.82±2.59^b^	5.67±0.52^d-g^	1.48±0.18^c^	0.73±0.20^h-k^	2.02±0.50^d-h^	1.38±0.02^i-l^	0.28±0.06^e-h^	0.23±0.01^i-l^	0.00±0^f^	2.30±1.04^ef^
**MM195**	7.60±1.71^n^	5.76±0.95^o^	19.87±3.40^m-o^	16.6±1.12^1o^	3.85±0.22^i-m^	2.85±0.45^m^	0.67±0.21^h-l^	0.45±0.12^kl^	1.79±0.27^f-i^	1.48±0.17^h-l^	0.34±0.09^bc^	0.21±0.06^j-l^	0.00±0^f^	1.44±0.12 ^h-k^
**MM684**	10.87±0.61^j-l^	9.13±0.64^n^	24.89±1.08^lm^	23.75±0.40^l-n^	3.69±0.56^j-m^	3.25±0.35^k-m^	0.77±0.06^f-k^	0.61±0.12^i-l^	1.82±0.31^e-i^	1.39±0.32^i-l^	0.31±0.03^c-e^	0.21±0.06^kl^	0.00±0^f^	2.07±0.04^ef^
**MM510**	11.00±0.53^jk^	8.93±0.50^mn^	28.75±0.82^kl^	26.252.72±^l^	4.36±0.19^g-k^	2.98±0.56^lm^	0.84±0.05^f-j^	0.39±0.08^l^	2.30±0.26^c-g^	1.57±0.45^h-k^	0.29±0.06^d-g^	0.21±0.03^kl^	0.00±0^f^	1.76±0.21^gh^
***S. sisybriifolium***	30.60±3.14^a^	23.80±3.30^b^	17.94±2.07^no^	15.311.79±^o^	7.59±0.31^bc^	5.72±1.20^d-g^	1.35±0.02^cd^	0.93±0.33^e-i^	4.11±0.16^a^	2.37±0.79^c-f^	0.36±0.03^ab^	0.24±0.08^h-k^	0.00±0^f^	1.41±0.08^h-k^
**TB**	15.60±0.87^fg^	12.40±0.35^ij^	40.98±1.36^h^	38.80±1.42^h-j^	5.82±0.81^d-f^	4.69±0.03^f-j^	1.08±0.35^d-f^	0.76±0.05^g-k^	2.39±0.36^c-e^	1.53±0.83^h-k^	0.26±0.02^g-i^	0.22±0.06^i-l^	0.00±0^f^	1.13±0.08^k^
**BB**	19.70±2.65^d^	14.50±3.87^gh^	44.04±3.42^gh^	39.01±3.98^h-j^	6.42±0.40^c-e^	5.34±0.52^e-h^	0.95±0.10^e-h^	0.73±0.12^h-k^	3.13±0.68^b^	1.53±1.04^h-k^	0.30±0.05^e-f^	0.22±0.08^i-l^	0.00±0^f^	1.52±0.26^h-i^
**MK**	22.93±0.85^bc^	20.33±0.47^d^	63.17±4.85^bc^	50.65±1.90^ef^	11.36±0.96^a^	8.77±0.52^b^	3.57±0.86^a^	2.10±0.77^b^	1.20±0.13^j-m^	0.63±0.01^m-n^	0.29±0.06^d-g^	0.17±0.01^m^	0.00±0^f^	1.64±0.17^g-j^
**AH**	22.53±1.00^bc^	15.37±0.95^f-h^	58.28±14.87^cd^	39.89±9.33^hi^	8.91±2.34^b^	5.33±1.83^e-h^	1.30±0.336^cd^	0.79±0.28^f-j^	1.04±0.20^k-m^	0.43±0.15^n^	0.19±0.01^lm^	0.13±0.06^n^	0.00±0^f^	1.15±0.04^jk^
**Topan 374**	21.27±2.42^cd^	17.70±2.03^e^	65.77±7.38^b^	56.51±2.94^de^	11.29±3.24^a^	8.18±1.36^b^	2.22±0.90^b^	1.18±0.43^c-e^	1.50±0.20^h-l^	0.94±0.50^l-n^	0.38±0.11^a^	0.20±0.11^lm^	0.00±0^f^	1.70±0.18^g-i^
**Kemer**	20.20±0.69^d^	15.73±1.47^fg^	48.30±5.86^fg^	42.152.72±^h^	6.32±0.67^c-e^	5.10±0.27^e-i^	0.86±0.23^f-j^	0.63±0.01^h-l^	2.69±0.81^bc^	1.33±0.54^i-l^	0.29±0.06^d-g^	0.19±0.07^lm^	0.00±0^f^	1.38±0.16^h-k^
**Amadeo**	13.73±0.42^hi^	11.60±0.11^jk^	42.01±5.01^h^	34.29±1.78^i-k^	5.61±0.45^d-h^	4.54±0.53^f-k^	0.81±0.17^i f-j^	0.63±0.05^-l^	1.85±0.21^e-i^	1.28±0.28^i-l^	0.27±0.09^f-h^	0.17±0.08^m^	0.00±0^f^	1.05±0.16^k^
**Faselis**	16.52±1.96^ef^	12.27±1.33^ij^	59.51±8.84^cd^	39.47±0.45^h-j^	5.35±5.36^e-h^	4.26±0.19^h-l^	1.21±0.56^c-e^	0.57±0.04^j-l^	2.47±0.51^cd^	1.49±0.41^h-l^	0.27±0.09^f-h^	0.22±0.03^i-l^	0.00±0^f^	1.26±0.07^i-k^
**Bildircin**	20.00±0.35^d^	16.27±0.87^ef^	40.32±6.86^hi^	33.82±1.38^jk^	6.75±0.38^cd^	5.41±0.53^d-h^	1.06±0.06^d-g^	0.83±0.23^f-j^	2.79±1.01^bc^	1.78±0.09^g-j^	0.32±0.06^b-d^	0.25±0.03^h-j^	0.00±0^f^	1.05±0.06^k^
**Genotype**	***		***		***		***		***		**		***	
**Treatment**	***		***		***		***		***		***		***	
**Genotype X Treatment**	***		***		*		***		*		**		***	
**CV%**	6.83		9.15		14.00		17.27		19.55		8.33		41.9	
**LSD**	1.74		6.04		1.36		0.32		0.62		0.04		0.5	

*: Data in columns with different letters indicate significant differences among samples at p < 0.05 by LSD multiple range test; ns: non significant; *, **, *** significant at p < 0.05, p < 0.01, and p < 0.001, respectively; CV, coefficient of variation; LSD, Least significant difference.

**Figure 2:**
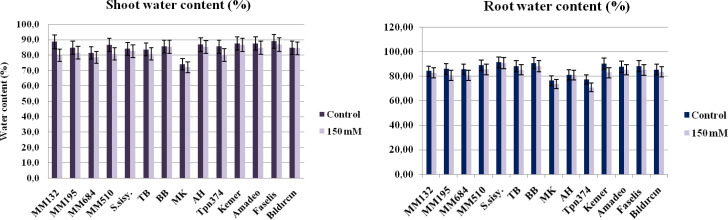
The shoot and root water contents of the genotypes subjected to salt stress by the 150-mM dose of NaCl (bars represent means ± standard errors).

Even though the water content of shoots and roots was decreased under salt stress, differences were released among the genotypes (**Figure 2**). The plant material that lost the most shoot moisture was found to be *S. macrocarpon* (10.1%). On the contrary, the BB local genotype showed the least variability in shoot water content compared to their control (0.2%). There were two studies in literature on the salt tolerance of *S. macrocarpon*, both of them using 0–30–60–90 and 120-mM NaCl doses, which discovered that the aerial parts were more sensitive to salinity than roots (Elisée et al., 2021), and salt stress reduces the growth due mainly to Na^+^ ion toxicity; moreover, the ratio of ionic selectivity (K^+^/Na^+^) played an important role rather than K^+^ ion content (Gildas Yénoukounmè Sounou et al., 2023).

**Figure 3:**
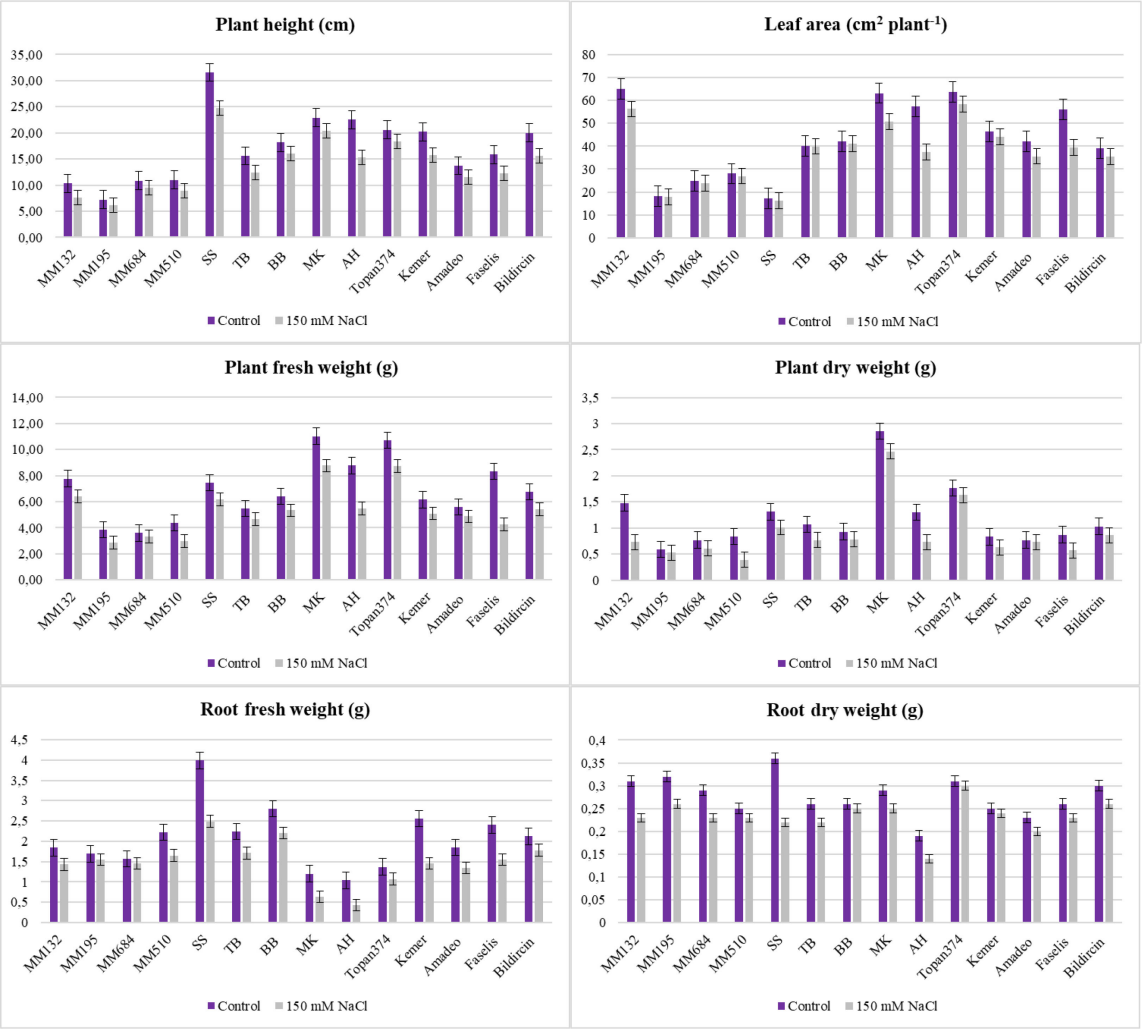
Plant height, leaf area, and fresh and dry weights of plants and roots of each 14 genotypes under salt stress (bars represent means ± standard errors).

Growth was inhibited by salt stress, and all the tested genotypes expressed different responses (*p* < 0.05, *p* < 0.01, or *p* < 0.001) with respect to each parameter studied in the experiment. Besides the effects of genotype and treatment individually, the interaction between them was also found significant (**Table 2**, **Figure 3**). The plant height, leaf area, shoot fresh/dry weight, and root fresh/dry weight of all genotypes were considerably reduced under salt stress effect. The length of each plant in the study was measured after treatment, and differences were found significant at *p* < 0.001. The wild relatives have species-specific development; therefore, *S. sisymbriifolium* (30.60 cm in control and 23.80 cm in salt stressed) and *S. linneanum* (7.60 cm in control and 5.76 cm in salt stressed) were defined as the longest and the shortest genotypes, respectively, in both control and stressed conditions. In addition, local genotype MK was distinguished with its remarkably long plants (20.33 cm) even under stressed conditions (**Table 2**, **Figure 3**). *S. sisymbriifolium* is known for its fast-developing, strong plants; thus, the obtained result was reasonable and genotypic differences were expected. For this reason, the percent change (%) values calculated and the least reduction in plant height revealed from MK, Amadeo, and *S. incanum* were 11.35%, 15.33%, and 16.51% respectively (**Supplementary Table S1**).

The lowest leaf area was measured from the wild relatives *S. sisymbriifolium* and *S. linnaeanum* at 15.31 cm^2^ plant^−1^ and 16. 61 cm^2^ plant^−1^, and the highest leaf area was measured from Topan374 and MK with 56.51 cm^2^ plant^−1^ and 50.65 cm^2^ plant^−1^ in the salt-treated group (**Table 2**, **Figure 3**). According to leaf areas, *S. sisymbriifolium* and *S. linnaeanum* were located in the same group; they both have more lobed leaves, which may cause their leaf area to be lower than the others. The lowest decrease in leaf area acquired from *S. incanum* was 4.82% (**Supplementary Table S1**).

While the highest shoot fresh and dry weights were obtained from MK as 8.77 g plant^−1^ and 2.10 g plant^−1^, respectively, the lowest shoot fresh and dry weights were weighed as 2.85 g plant^−1^ and 0.39 g plant^−1^ from wild relatives *S. linnaeanum* and *S. insanum*, respectively. In terms of percent change, *S. incanum* (10.81% for SFW and 25% for SDW) and BB (17.19% for SFW and 22.22% for SDW) genotypes showed the least change for both shoot fresh and dry weights (**Supplementary Table S1**). Abbas et al. (2010) reported that 100 mM NaCl (EC of 10 dS m^−1^) stress noticeably suppressed the shoot length and shoot fresh and dry weights of both eggplant cultivars Bemisal and Dilnasheen. However, cv. Bemisal was comparatively better in these traits than cv. Dilnasheen. Compatible with the literature, extensive differences in salt tolerance have been observed among the employed genotypes and accessions. The genotype AH has the lowest (0.43 g–0.13 g) root fresh and dry weights among the accessions whereas the least reduction was acquired from TB (15.38%) for these parameters (**Supplementary Table S1**). These results proved that besides some employed CWRs, except AH, local genotypes were found prominent with their root characteristics (**Table 2**, **Figure 3**).

**Figure 4:**
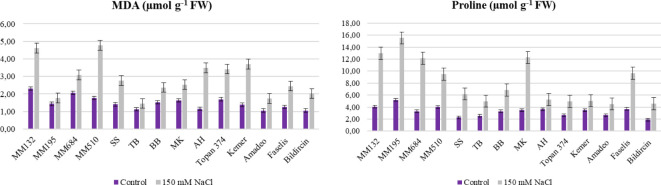
MDA and proline content of salt treated and control plants of 14 genotypes (Bars represent means ± standard errors).

In this study, MDA accumulation in leaves was determined for both control and salt-stressed plants and the results indicated highly significant differences. Among the plant material, MDA accumulation ranged from 1.46 µmol g^−1^ FW (TB) to 4.61 µmol g^−1^ FW (**Figure 4**, **Table 3**). Increasing of MDA level in leaves means decrease of tolerance, and the minimum increase was acquired from *S. linnaeanum* (23.82%) and MK (28.77%) (**Supplementary Table S1**). Plants under stress also accumulate proline, and high accumulation indicates high tolerance. Wild crop relative *S. linnaeanum* (15.53 µmol g^−1^) was distinguished the most proline accumulator and MK (12.27 µmol g^−1^ FW) with *S. incanum* (12.16 µmol g^−1^ FW) followed after *S. linnaeanum* (**Figure 4**, **Table 3**). Moreover, a maximum proline increase was obtained from *S. incanum* (268.97%), MK (253.15%), and *S. linnaeanum* (198.70%) (**Supplementary Table S1**).

**Table 3:** Biochemical evaluation of eggplant genotypes at the 12^th^ day of the 150 mM NaCl treatment*

Genotypes	Na-shoot(%)		Ca-shoot(%)		K-shoot(%)		Na-root(%)		Ca-root(%)		K-root(%)		MDA (µ mol g^-1^ FW)		Proline (µ mol g^-1^ FW)	
	Control	150 mM NaCl	Control	150 mM NaCl	Control	150 mM NaCl	Control	150 mM NaCl	Control	150 mM NaCl	Control	150 mM NaCl	Control	150 mM NaCl	Control	150 mM NaCl
**MM132**	0.14±0.05^k^	3.69±0.51^b^	3.33±0.40^c^	1.90±0.64^h-j^	5.46±0.85^a^	3.41±2.31^fg^	0.27±0.14^l-n^	1.43±0.51^e^	3.97±0.79^a^	1.93±0.82^c^	5.07±0.35^a^	2.86±0.89^fg^	4.61±0.55^a^	3.36±1.12^f-j^	8.82±2.16^c^	1.20±0.53^c-e^
**MM195**	0.11±0.01^k^	1.18±0.17^f^	0.45±0.07^op^	0.39±0.06^p^	2.16±0.21^l^	2.03±0.33^l-h^	0.83±0.04^hi^	1.66±0.15^d^	0.36±0.36^k-m^	0.26±0.02^lm^	1.96±0.19^h-l^	1.81±0.18^j-l^	1.78±0.96^gh^	5.20±1.18^d-g^	15.53±5.08^a^	0.27±0.12^f^
**MM684**	0.04±0.02^k^	0.76±0.49^g-i^	3.24±0.01^c^	2.44±0.27^e-g^	2.76±0.08^i-k^	2.26±0.29^l^	0.48±0.01^j^	1.38±0.25^ef^	1.44±0.06^d-g^	1.26±0.74^f-i^	2.08±0.12^h-l^	1.77±0.31^kl^	3.09±0.22^c^	3.30±0.95^g-j^	12.16±0.87^b^	1.67±0.64^bc^
**MM510**	0.10±0.06^k^	2.29±0.18^cd^	5.37±0.16^a^	1.08±0.63^m-n^	3.18±0.28^g-i^	1.53±0.65^m^	0.10±0.04^o^	0.84±0.21^hi^	1.62±0.55^de^	0.53±0.89^kl^	2.39±0.22^g-j^	1.91±0.32^i-l^	3.01±0.15^cd^	4.02±0.45^f-i^	9.48±0.92^c^	3.20±0.20^a^
***S. sisymbriifolium***	0.13±0.10^k^	2.39±0.35^cd^	4.43±0.62^b^	2.55±0.37^de^	5.46±0.45^a^	3.40±0.49^f-h^	0.30±0.27^k-n^	2.57±0.08^a^	3.32±1.19^b^	1.69±1.17^cd^	3.38±0.60^d-f^	2.53±0.33^gh^	4.34±0.39^a^	3.95±0.90^f-i^	6.20±0.60^de^	1.53±0.42^b-d^
**TB**	0.38±0.56^i-k^	3.47±0.91^b^	2.13±0.13^e-h^	1.98±0.12^g-j^	4.78±0.20^b^	4.05±0.13^c-e^	0.14±0.02^no^	1.04±0.07^g^	0.50±0.12^k-m^	0.28±0.12^k-m^	4.19±1.41^b^	3.61±0.92^b-e^	1.46±0.06^h-k^	2.54±0.52^ij^	4.99±0.99^d-g^	0.93±0.31^e^
**BB**	0.09±0.15^k^	0.88±0.40^f-h^	1.09±1.74^l-n^	0.90±0.21^m-o^	2.43±0.41^j-l^	2.27±0.09^kl^	0.86±0.01^hi^	1.25±0.12^f^	0.54±0.02^k^	0.42±0.04^k-m^	2.31±0.32^g-l^	2.00±0.22^h-l^	2.37±0.21^ef^	3.30±0.78^g-j^	6.83±0.40^d^	1.00±0^e^
**MK**	0.61±0.41^h-j^	3.51±0.11^b^	2.49±0.12^d-f^	2.04±0.04^f-i^	4.29±0.14^b-d^	3.58±0.20^e-g^	0.43±0.06^jk^	0.91±0.02^g-i^	1.59±0.01^de^	1.37±0.03^e-h^	3.90±0.07^b-d^	3.25±0.08^ef^	2.53±0.28^de^	3.47±0.40^f-j^	12.27±0.83^b^	1.06±0.87^de^
**AH**	0.17±0.02^k^	4.62±1.25^a^	1.78±0.15^h-k^	1.03±0.54^l-n^	2.91±0.71^h-j^	2.21±0.10^l^	0.30±0.03^k-n^	1.29±1.02^ef^	1.56±0.01^de^	1.12±0.25^hi^	2.43±0.05^g-i^	1.76±0.19^l^	3.49±0.17^bc^	3.64±0.37^f-j^	5.24±0.72^d-f^	3.07±0.70^a^
**Topan 374**	0.15±0.55^k^	2.65±1.05^c^	1.83±1.73^h-j^	0.63±0.41^n-p^	4.58±0.85^b^	3.57±0.22^e-g^	0.20±0.30^m-o^	0.99±0.52^gh^	1.47±0.29^d-f^	1.27±0.59^f-i^	3.46±0.31^c-f^	2.54±0.78^gh^	3.42±0.33^bc^	2.66±1.42^h-j^	4.97±0.80^d-g^	2.00±0.35^b^
**Kemer**	0.06±0.71^k^	1.13±0.55^fg^	0.63±0.24^n-p^	0.44±1.11^op^	4.49±0.9^bc^	3.19±0.49^g-i^	0.33±0.05^j-m^	1.86±0.60^c^	0.36±0.17^k-m^	0.23±0.15^m^	2.38±0.56^g-k^	1.80±0.68^j-l^	3.71±0.13^b^	3.49±0.77^f-j^	5.06±0.42^d-g^	1.07±0.12^de^
**Amadeo**	0.08±0.09^k^	2.12±0.54^de^	1.50±0.09^j-l^	1.34±0.20^k-m^	3.77±0.78^ef^	3.51±0.56^fg^	0.75±0.07^i^	2.43±0.60^a^	1.16±0.09^g-i^	1.06±0.15^ij^	4.00±2.90^bc^	3.32±0.59^d-f^	1.74±0.05^g-i^	2.66±0.92^h-j^	4.52±0.44^e-h^	0.93±0.31^e^
**Faselis**	0.10±0.33^k^	2.53±1.32^c^	4.16±0.42^b^	1.62±0.23^i-k^	3.24±0.39^g-i^	2.30±0.41^kl^	0.40±0.17^j-l^	2.14±0.40^b^	1.42±0.18^d-g^	0.83±0.39^j^	3.61±2.90^b-e^	2.41±0.59^g-j^	2.45±0.41^ef^	3.70±0.92^f-j^	9.65±0.89^c^	1.00±0.20^e^
**Bildircin**	0.24±0.25^jk^	1.79±0.99^e^	2.97±0.57^cd^	2.50±1.79^d-f^	3.84±0.41^d-f^	3.44±0.74^fg^	0.43±0.02^j-l^	1.24±0.133^f^	1.57±0.34^de^	1.38±0.17^e-h^	3.61±0.12^b-e^	3.19±0.52^ef^	2.03±0.17^fg^	1.92±0.31^j^	4.57±0.72^e-g^	1.00±0^e^
**Genotype**	***		***		***		***		***		***		***		***	
**Treatment**	***		***		***		***		***		***		***		***	
**Genotypes × Treatment**	***		***		***		***		***		*		***		***	
**CV%**	19.84		15		8.93		10.42		13.82		13.38		14.29		20.64	
**LSD**	0.42		0.5		0.48		0.16		0.28		0.62		0.5		1.9	

*: Data in columns with different letters indicate significant differences among samples at p < 0.05 by LSD multiple range test; ns: non significant; *, **, *** significant at p < 0.05, p < 0.01, and p < 0.001, respectively; CV, coefficient of variation; LSD, Least significant difference.

**Figure 5:**
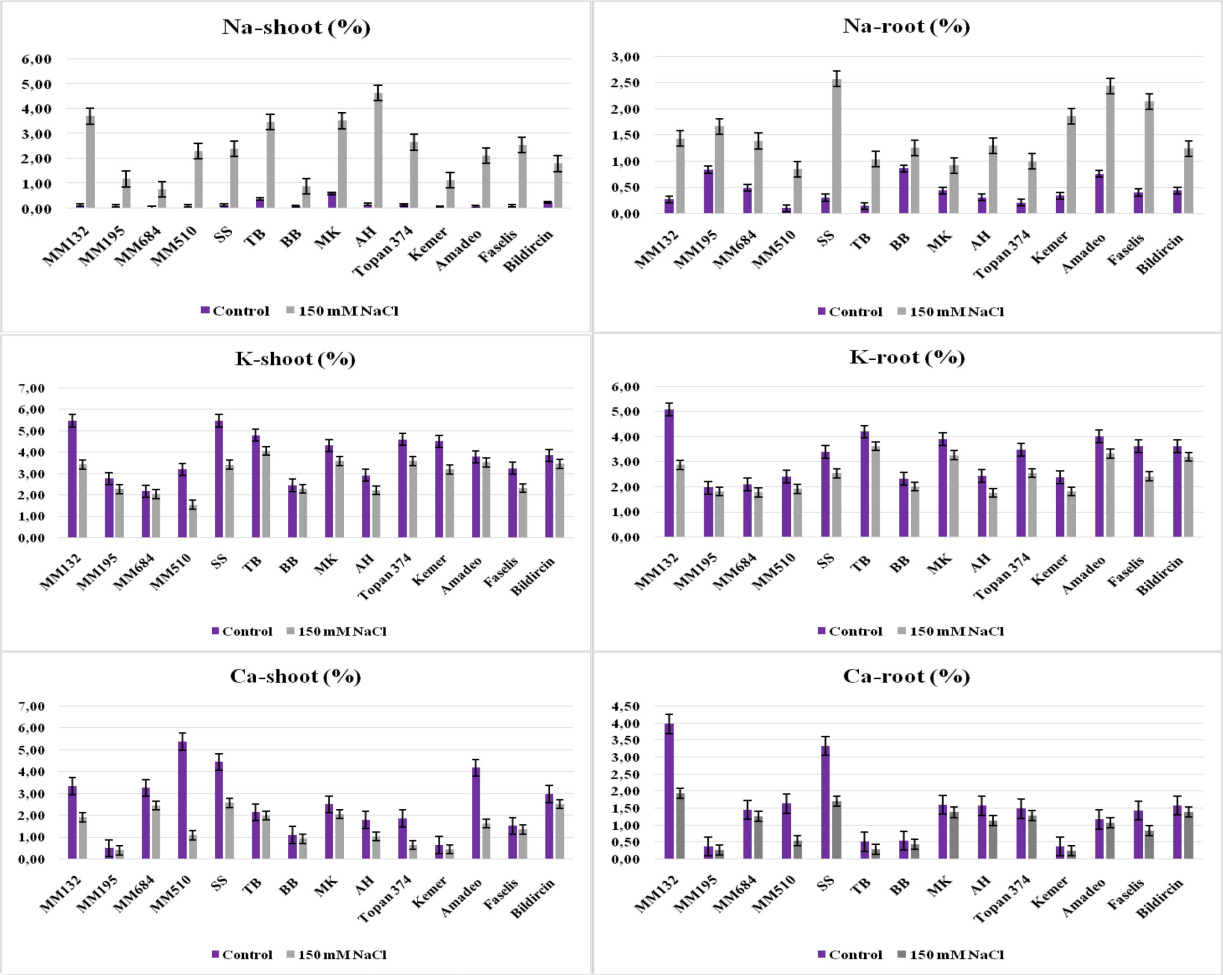
The Na^+^, K^+^, and Ca^2+^ accumulation in shoot and root of salt-treated and control plants of 14 genotypes (bars represent means ± standard errors).

In terms of ion accumulation, while the sodium (Na^+^) in root and shoot of salt-treated plants increased, potassium (K^+^) and calcium (Ca^+2^) levels decreased. Suarez et al. (2021) declared that the high salt tolerance level of eggplant is related to the low Na^+^ concentration in leaves. It was obvious from the study that wild relatives *S. incanum* and *S. linneanum*, local genotype BB, and Kemer variety have the least Na^+^ amount in their leaves, whose Na^+^ transport from roots to shoots was restricted under the salt effect (**Table 3**, **Figure 5**). Akinci et al. (2004) reported that the Kemer variety was tolerant to salt stress, which was applied with 50-mM and 150-mM NaCl doses in their research. While the salt tolerance level of *S. linneanum* was studied for the first time in this research, the salt tolerance of *S. incanum* (Cebeci et al., 2024) and BB (Yasar et al., 2013) was as previously reported. The highest Na^+^, Ca^2+^, and K^+^ accumulations in roots were recorded from *S. sisymbriifolium*, *S. macrocarpon*, and TB, respectively (**Table 3**).

Suarez et al. (2021) reported that the K^+^-salinity/K^+^-control ratio of a leaf was significant in finding the genotype tolerance level under NaCl stress. The least reduction of K^+^ concentration in shoots was measured from *S. linneanum* as 6.16%, BB as 6.58%, and Amadeo as 6.81% (**Figure 5**, **Supplementary Table S1**). Analysis of Ca^+2^ concentrations from shoots revealed that the lowest decreases were obtained from local genotypes TB (7.05%), BB (17.27%), and MK (17.83%) (**Table 3**, **Figure 5**).

**Figure 6:**
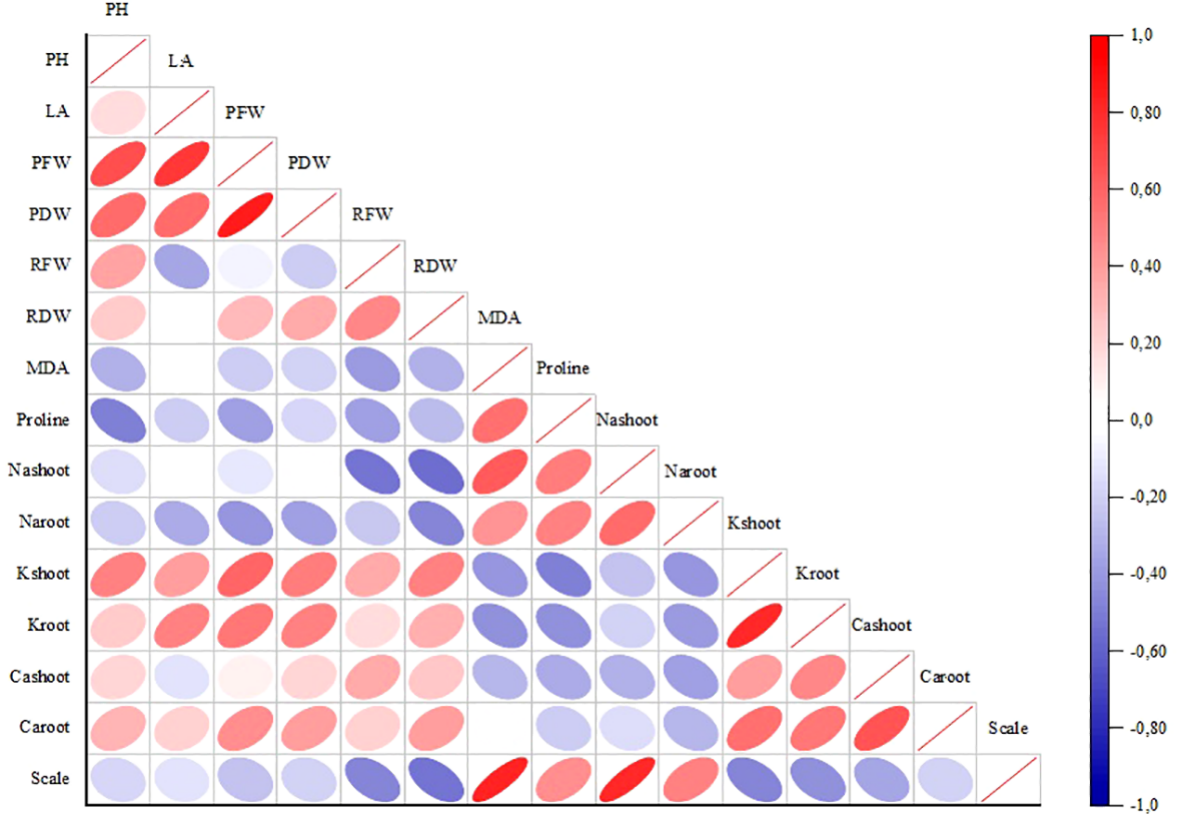
Correlation analysis between growth and physiological parameters. PH, plant height; LA, leaf area; PFW, plant fresh weight; PDW, plant dry weight; RFW, root fresh weight; RDW, root dry weight.

The graph in **Figure 6** displays the association of employed morphologic and biochemical features. The thickness of the ellipses in squares means the level of correlation. Accordingly, strong positive correlations were detected between SFW (mentioned in the graph as plant fresh weight (PFW)), PH, SDW (mentioned in the graph as plant dry weight (PDW)), and PH, LA. Positive correlations were remarkable between scale and MDA, Na-shoot. On the other hand, the correlations between proline and PH, Na-shoot and RFW, RDW were found negative.

**Figure 7:**
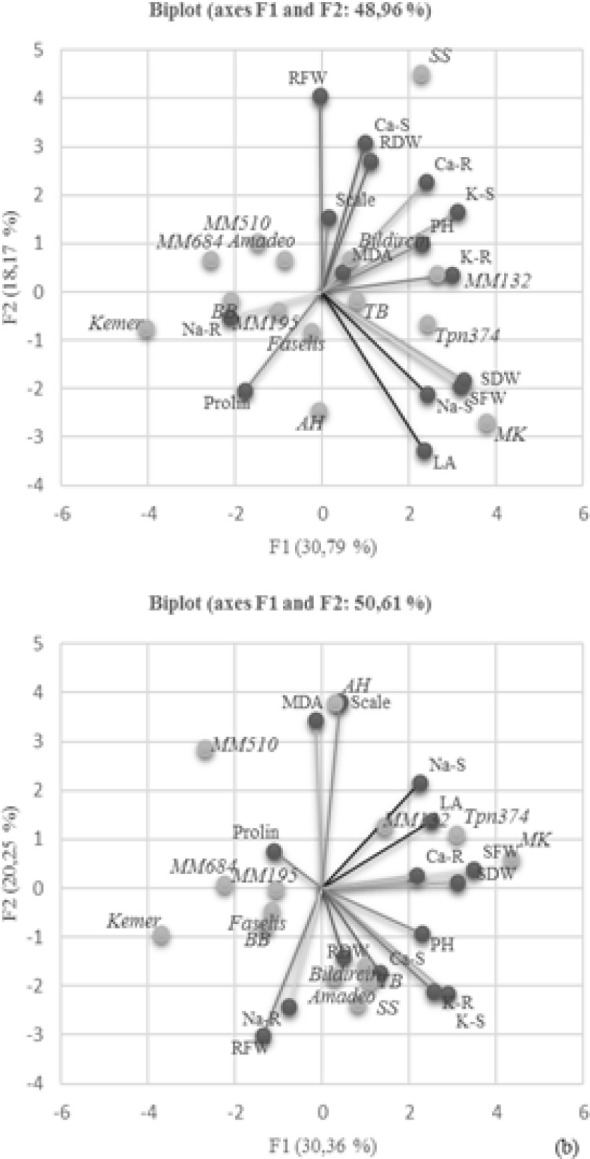
Distribution of the investigated parameters and employed genotypes in principal coordinates Bi-plot–control **(A)** and Bi-plot–salt stress **(B)**. PH, plant height; LA, leaf area; SFW, mentioned in the graph as plant fresh weight (PFW); SDW, mentioned in the graph as plant dry weight (PDW); RFW, root fresh weight; RDW, root dry weight; S, shoot; R, root.

Principal component analysis (PCA) was performed to clear which parameter is more effective in explaining the variation. The distributions of both genotypes and parameters were examined on the coordinate plane for control (**Figure 7A**) and salt treatment (**Figure 7B**). The origin point on the coordinate plane is the area with the least variation; the close positioning of the parameters or genotypes indicates that there are many similarities between them. With respect to Bi-plot analysis, the first two components accounted for 48.96% of the total variation for control and 50.61% for salt stress (**Figures 7A, B**). The Bi-plot revealed a strong correlation between proline accumulation and *S. linneanum* and between MDA accumulation and AH local genotype under salt treated conditions.

**Figure 8:**
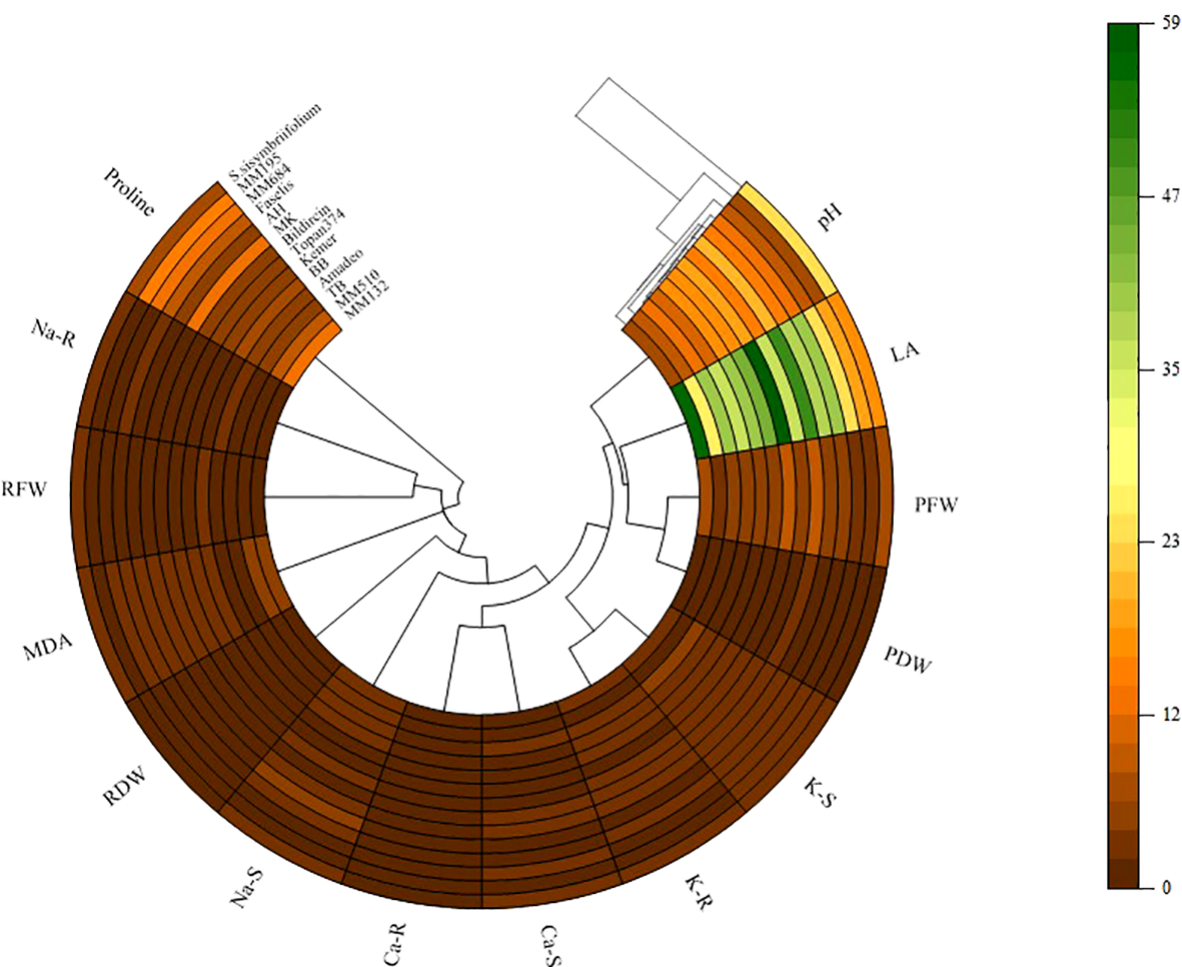
Polar heatmap with dendrogram between growth and physiological parameters. PH, plant height; LA, leaf area; SFW, mentioned in the graph as plant fresh weight (PFW); SDW, mentioned in the graph as plant dry weight (PDW); RFW, root fresh weight; RDW, root dry weight; S, shoot; R, root.

The polar heat map in **Figure 8** was created based on correlation data related to the variables examined. The relationship between eggplant genotypes and growth parameters, ion contents, and biochemical variables showed more differences under salt stress. It was observed that genotypes generally separated into different groups under salt stress. While RDW and RFW demonstrated direct clustering with genotypes, indirect clustering was noted in root and shoot ion accumulations.

## 4 Discussion

In the current study, wild and cultivar forms of eggplant was examined by using plant growth and biochemical parameters under salt stressed conditions at seedling stage. In general, tolerance against salt stress increases as the plant matures and plants at germination and seedling stages represent more damages to salinity stress; thus, determination of salt tolerance of the genotypes at these stages is significant (Akinci et al., 2004). The salinity tolerance of eggplants may vary according to their genetic bases (Lima et al., 2015; Oliveira et al., 2016) considering the employed plant material and previous studies; 150 mM NaCl-based saline stress was preferred to reveal potential salt-tolerant genotypes from different resources. In tomato and eggplant, Na^+^ is the major harmful ion and sensitivity to Na^+^ was linked to the plant’s inability to limit Na^+^ uptake and transfer to the leaf (Suarez et al., 2021).

Dash and Panda (2001) reported that one of the earliest responses to the salt stress is leaf area reduction, and genotypes exhibited a reduction in different degrees according to their growth habit. Consistent with the previous studies, parameters like leaf area, plant height, and shoot and root fresh/dry weights were decreased under the salt effect. In a study, leaf dry weight and leaf area of *Solanum scabrum* Mill. and *Solanum melongena* L. were reduced by 25%–18% and 47%–55%, respectively, with the application of 50 mM NaCl (Assaha et al., 2013). The highest leaf area was obtained from Topan374 (56.51 cm) and one of the lowest values obtained from *S. linneanum* (16.61 cm); however, the integrated results considered *S. linneanum* to be more tolerant compared with Topan374. In this case, percent change (%) values become more explanatory (**Supplementary Table S1**). This is because in terms of percent change (%), as well as *S. incanum*, TB, BB, and *S. linneanum* also presented one of the least leaf area reductions as a response to the salt effect. It was clear from the present study that distant relatives have species-specific growth features; thus, the plant height of salt-sensitive *S. sisymbrifolium* measured longer than the salt-tolerant *S. linneanum*. Comparison of their tolerance level considering plant height becomes not suitable. However, in a study where the salt tolerance level of the eggplant F_2_-segregating population was investigated, plant height was found to be a very useful separator (Cebeci et al., 2024). In addition to this, decreases in plant height due to the increase in salt concentration are compatible with the previous studies conducted by various researchers (Akinci et al., 2004; Unlukara et al., 2010; Kiran et al., 2015; Brenes et al., 2020a; Brenes et al., 2020b).

Leaf Na^+^ content and plant tolerance index had a negative correlation (Raza et al., 2017). Salt stress effects on plant metabolism have been related to the K^+^ and Ca^2+^ ion depletion and toxic ions like Na^+^ and Cl^−^ accumulation in leaves (Munns and Tester, 2008). Assaha et al. (2013) reported that the increases in leaf sodium (Na^+^) accumulation of cultivar eggplant and wild relative *Solanum scabrum* Mill were found to be 65 and 66%–18% and 36%, respectively, under 50-mM and 150-mM salt treatments. In the present study, a 150-mM dose of NaCl application caused increases in Na^+^ accumulation in shoots and roots (**Supplementary Table S1**); the genotypes have the least increase (MK with 478.02% in shoots and BB with 46.30%, *S. linneanum* with 99.60% in roots), which were determined putative and salt tolerant. The lowest concentration of Na^+^ was accumulated in the roots of BB, *S. linneanum*, and MK under salt stress, and the transfer of Na^+^ from their root to shoot was also found very limited. Genotypes that give these similar responses may activate some Na^+^ exclusion mechanisms, such as Na^+^ flow from roots to rhizosphere and Na^+^ loading and unloading in xylem, and tend to have a low amount of Na^+^ in leaves to keep plants alive by showing high tolerance to saline environments (Ismail and Horie, 2017). Moreover, high accumulations of Na^+^ in leaf resulted in increases in Na^+^/Ca^2+^ and Na^+^/K^+^ ratios, which caused a decrease in total chlorophyll content and drastic reduction in growth parameters (Assaha et al., 2013; Hannachi and Van Labeke, 2018). In terms of percent change calculations, *S. linnaeanum* was distinguished as the highest accumulator of the K^+^ and Ca^2+^ ions in roots and shoots under stress, followed by the BB genotype (**Supplementary Table S1**). *S. linnaeanum* continued the water uptake under salt stress by increasing the osmotic pressure of the shoot cells, thanks to more K^+^ ions it received through active ion uptake from the saline soil. Similar performance was observed in *S. melongena* and *Solanum torvum* plants by Brenes et al. (2020a). The enhanced leaf Ca^2+^ accumulation is believed to have contributed to the resistance to salt stress (Assaha et al., 2013) and has an effect on alleviating the negative effects of stress (Chen et al., 2016) in eggplant. A decrease in the Ca^2+^ amount was detected to be low in local genotypes TB, MK, and BB in shoots and commercial varieties Amadeo and Topan374 in roots (**Supplementary Table S1**). Among the local genotypes included in this trial, AH showed sensitivity to salt stress; previously it was tested by Talhouni et al. (2019), and consistent with the present study, similar results were reported.

The lowest MDA increases were observed in *S. linnaeanum* (23.82%), TB (28.77%), and *S. incanum* (49.30%) genotypes in the experiment. High enzymatic activity suggests more tolerance to salinity (Yasar et al., 2013); hence, CWRs produce higher antioxidant enzymes and less MDA under salt stress conditions in the experiment. Previous studies on eggplant proved that plants with high proline accumulation displayed greater tolerance to stress conditions (Plazas et al., 2019). Interestingly, proline contents of *S. linnaeanum*, *S. incanum*, and MK were also detected to be higher than the other genotypes and accessions. It is known that proline has antioxidant properties and increases the plants’ tolerance to stressed conditions, as it can act as a molecular chaperone protein to protect the structure of biological macromolecules during dehydration (Ashraf and Foolad, 2007; Tang et al., 2015). Additionally, principal component analysis also revealed a strong correlation between proline accumulation–*S. linneanum* accession and MDA accumulation–AH local genotype under salt-treated conditions. Correlation analysis proved that there are positive correlations between scale and MDA, Na-shoot values.

In previous studies, MK, AH, and BB genotypes were tested against salt stress in *in vivo* and *in vitro* conditions, and while MK and BB genotypes were stated as tolerant, the AH genotype was labeled as sensitive (Yasar et al., 2013). Likewise, MK and BB genotypes were found tolerant compared with the AH genotype in the study. However, wild relative *S. linneanum* presented better performance than these local genotypes under salt stress. According to Zurayk et al. (1993), there is more than one mechanism responsible for salinity tolerance in eggplant. Therefore, diverse varietal responses under salt stress may be due to the different tolerance mechanisms of the used plant materials. They also reported that some local genotypes performed better under salt stress than commercial ones. Similar results were obtained in the present study.

## 5 Conclusion

Comprehensive research was carried out to find the most salt-tolerant candidate lines using different genetic sources including wild, commercial, and local genotypes. The phenotypically observed differences were associated with metabolic changes, and with the evaluation of percent change values, plant materials which were sourced from different ecologies were compared with each other. Wild relatives have species-specific growth features; thus, the salt tolerance levels of morphologic features such as plant height and leaf area were found inappropriate to be compared. Proline, MDA, and ion analyses of the experiment were compatible to each other, such as *S. linnaeanum* which was found to be the highest proline and lowest MDA accumulator. Similarly, low Na^+^ concentrations in roots of BB and *S. linneanum* caused high K^+^ and Ca^2+^ concentrations in leaves. Principal component analysis revealed strong correlations between proline accumulation–*S. linnaeanum* and MDA accumulation–AH genotype. Consequently, plenty of information has been gathered on responses of employed genotypes under salt stress. In terms of results, the most salt tolerant genotypes will be employed in future breeding studies to improve salt tolerant inbred lines and varieties through interspecific hybridization.

## Data Availability

The datasets presented in this study can be found in online repositories. The names of the repository/repositories and accession number(s) can be found in the article/**Supplementary Material**.
