# Supplementary material for: Comprehensive assessment to reveal the salt tolerance potential of cultivated eggplants and their wild relatives
Source: Front Plant Sci. 2025 Jan 30;16:1483409. doi: 10.3389/fpls.2025.1483409 (PMC11821942; doi:10.3389/fpls.2025.1483409)
Supplement: Supplementary file 1 [file DataSheet1.docx]

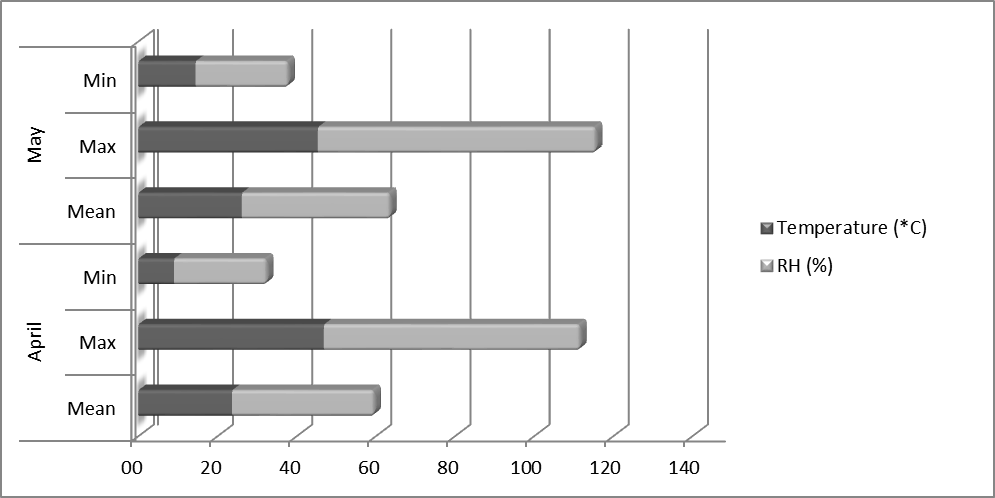


**Supplementary Figure 1.** The recorded maximum, minimum and mean values of temperature (^°^C) and relative humidity (%) from experimental area.


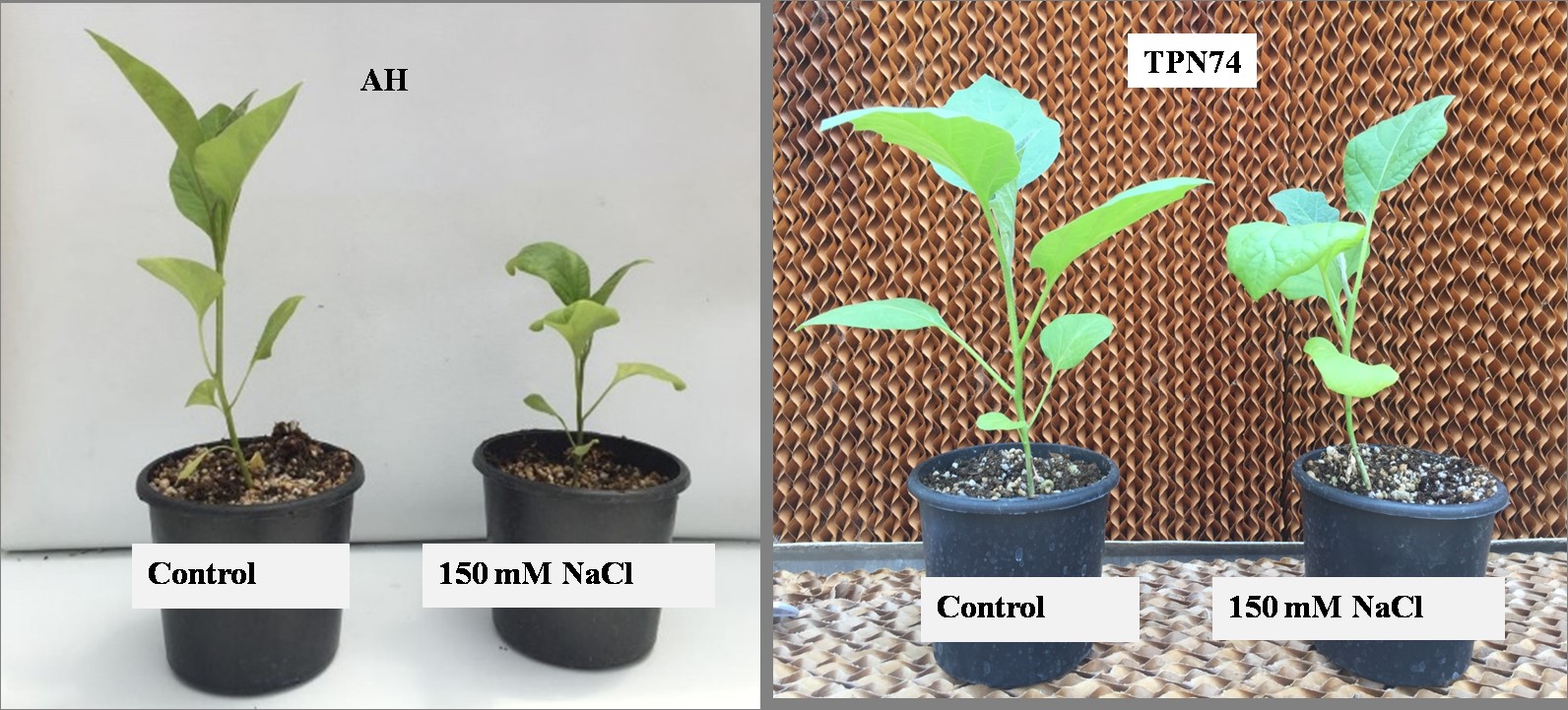


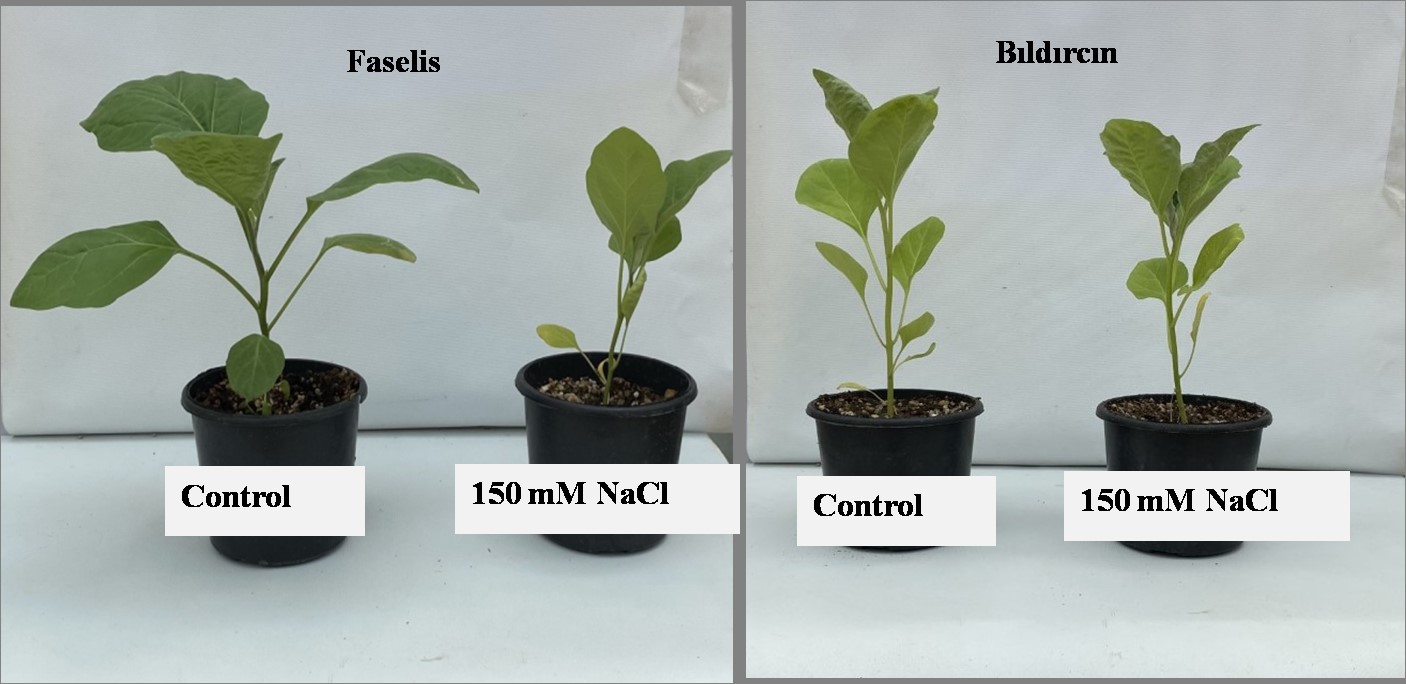


**Supplementary Figure 2.** Comparison of used genotypes under control and salt stressed conditions

**Supplementary Data Information**

The percent changes in the morphological and biochemical parameters induced by salt treatment were displayed variations in salt stress tolerance of eggplant genotypes. The percent changes in all growth parameters were correlated negatively in salt treated genotypes. The salt stress caused maximum reductions in shoot height as -31.80%, in leaf area as -25.23%, in root fresh weight as -58.33% and in root dry weight as -51.72% in the genotype AH. Similarly, the high reduction was recorded in accession *S. macrocarpon* for same parameters. However, the maximum reductions in shoot fresh weight with -48.98% in commercial genotype Faselis and shoot dry weight with -53.17% in accession *S. sisymbriifolium* were observed. Salt stress caused a significant increase in MDA content for all plant materials. The percent change was found above +200% especially for *S. sisymbriifolium* and AH. On the other hand, the percent change in proline for these genotypes was found quite low compared to other genotypes. In the current study, the great differences were detected in proline accumulation under salt stress condition. The highest percent change in leaf proline was recorded in *S. incanum*, MK, and *S. linneanum*. In addition to this, the changes in percent rates for Na^+^ ion content of genotypes were higher than the changes in other used parameters. Despite the Na^+^ ion concentration change was occurred as an increase in both shoot and root these changes were occurred as a decrease for K^+^ and Ca^+2^ ions (**Supplementary Table 1**).

**Supplementary Table 1.**The percent changes for morphologic and biochemical parameters of 14 eggplant genotypes subjected to salt stress

| **Genotypes** | **PH**  **(cm)** | **LA**  **(cm^2^plant^-1^)** | **SFW**  **(g plant^-1^)** | **SDW**  **(g plant^-1^)** | **RFW**  **(g plant^-1^)** | **RDW**  **(g plant^-1^)** | **MDA**  **(µ mol g^-1^ FW)** | **Proline**  **(µ molg^-1^ FW)** | **Na-shoot**  **%)** | **Na-root**  **%)** | **K-shoot**  **%)** | **K-root**  **%)** | **Ca-shoot**  **%)** | **Ca-root**  **%)** |
| --- | --- | --- | --- | --- | --- | --- | --- | --- | --- | --- | --- | --- | --- | --- |
| **MM132** | -29,52 | -31,43 | -35,23 | -53,33 | -30,00 | -17,86 | 100.33 | 162.67 | 2600.00 | 435.00 | -37.52 | -43.52 | -42.89 | -50.13 |
| **MM195** | -23,68 | -16,58 | -25,64 | -28,57 | -16,67 | -38,24 | 23.82 | 198.70 | 1009.38 | 99.60 | -6.16 | -7.48 | -20.00 | -26.32 |
| **MM684** | -16,51 | -4,82 | -10,81 | -25,00 | -22,22 | -32,26 | 49.30 | 268.97 | 1653.85 | 186.21 | -18.02 | -14.90 | -24.59 | -27.27 |
| **MM510** | -19,09 | -8,71 | -31,82 | -50,00 | -30,43 | -27,59 | 70.75 | 135.82 | 2193.33 | 768.97 | -51.99 | -20.06 | -79.91 | -73.32 |
| ***S. sisybriifolium*** | -22,22 | -14,53 | -25,00 | -35,71 | -41,46 | -33,33 | 207.17 | 57.07 | 1741.03 | 766.29 | -37.73 | -25.05 | -42.32 | -44.79 |
| **TB** | -20,51 | -5,37 | -18,97 | -27,27 | -37,50 | -15,38 | 28.77 | 96.62 | 821.24 | 657.32 | -15.27 | -13.84 | -7.05 | -43.40 |
| **BB** | -26,40 | -11,36 | -17,19 | -22,22 | -51,61 | -26,67 | 54.76 | 106.98 | 918.46 | 46.30 | -6.58 | -13.40 | -17.27 | -25.37 |
| **MK** | -11,35 | -19,78 | -22,81 | -41,67 | -50,00 | -41,38 | 54.79 | 253.15 | 478.02 | 110.00 | -16.63 | -16.58 | -17.83 | -20.69 |
| **AH** | -31,56 | -31,56 | -40,45 | -38,46 | -60,00 | -31,58 | 202.76 | 44.13 | 2670.00 | 330.00 | -23.97 | -27.71 | -42.13 | -23.61 |
| **Topan374** | -16,90 | -14,13 | -27,43 | -45,45 | -40,00 | -47,37 | 101.52 | 87.07 | 1709.09 | 386.89 | -22.11 | -26.66 | -65.42 | -7.59 |
| **Kemer** | -22,28 | -12,63 | -19,05 | -33,33 | -51,85 | -34,48 | 168.07 | 44.92 | 1777.78 | 457.00 | -29.03 | -24.23 | -30.53 | -36.11 |
| **Amadeo** | -15,33 | -18,33 | -19,64 | -25,00 | -31,58 | -37,04 | 64.83 | 70.11 | 2545.83 | 222.57 | -6.81 | -17.08 | -11.09 | -7.76 |
| **Faselis** | -25,45 | -33,61 | -20,37 | -50,00 | -40,00 | -18,52 | 94.37 | 160.90 | 2351.61 | 438.66 | -28.94 | -33.33 | -61.17 | -50.40 |
| **Bildircin** | -18,50 | -16,13 | -19,40 | -27,27 | -35,71 | -21,88 | 92.05 | 138.11 | 647.22 | 187.60 | -10.49 | -11.73 | -15.73 | -21.08 |

**SL:** Shoot length. **LA:** Leaf area. **SFW:** Shoot fresh weight. **RFW:** Root fresh weight. **SDW:** Shoot dry weight. **RDW:** Root dry weight. **MDA:**Malondialdehyde. **FW:** Fresh weight
